# Cyanidin-3-O-Glucoside Modulates the In Vitro Inflammatory Crosstalk between Intestinal Epithelial and Endothelial Cells

**DOI:** 10.1155/2017/3454023

**Published:** 2017-03-08

**Authors:** Daniela Ferrari, Francesco Cimino, Deborah Fratantonio, Maria Sofia Molonia, Romina Bashllari, Rossana Busà, Antonella Saija, Antonio Speciale

**Affiliations:** Department of Chemical, Biological, Pharmaceutical and Environmental Sciences, University of Messina, 98168 Messina, Italy

## Abstract

Intestinal epithelium represents a protective physical barrier and actively contributes to the mucosal immune system. Polarized basolateral intestinal secretion of inflammatory mediators, followed by activation of NF-*κ*B signaling and inflammatory pathways in endothelial cells, efficiently triggers extravasation of neutrophils from the vasculature, therefore contributing to the development and maintenance of intestinal inflammation. Proper regulation of NF-*κ*B activation at the epithelial interface is crucial for the maintenance of physiological tissue homeostasis. Many papers reported that anthocyanins, a group of compounds belonging to flavonoids, possess anti-inflammatory effects and modulate NF-*κ*B activity. In this study, by using a coculture in vitro system, we aimed to evaluate the effects of TNF-*α*-stimulated intestinal cells on endothelial cells activation, as well as the protective effects of cyanidin-3-glucoside (C3G). In this model, TNF-*α* induced nuclear translocation of NF-*κ*B and TNF-*α* and IL-8 gene expression in Caco-2 cells, whereas C3G pretreatment dose-dependently reduced these effects. Furthermore, TNF-*α*-stimulated Caco-2 cells induced endothelial cells activation with increased E-selectin and VCAM-1 mRNA, leukocyte adhesion, and NF-*κ*B levels in HUVECs, which were inhibited by C3G. We demonstrated that selective inhibition of the NF-*κ*B pathway in epithelial cells represents the main mechanism by which C3G exerts these protective effects. Thus, anthocyanins could contribute to the management of chronic gut inflammatory diseases.

## 1. Introduction

The intestinal epithelium represents protective physical barrier acting also through immune mechanisms. When intestinal epithelium is challenged by proinflammatory mediators such as TNF-*α* and interferon-*γ*, local communication between the gut epithelium and the endothelium of underlying blood vessels is needed to mount an effective and coordinated cellular immune response. Polarized secretion of chemoattractants promotes leukocyte recruitment and accumulation, by extravasation from the circulation through the local endothelium. In particular, IL-8 secretion, caused by polarized TNF-*α* stimulation in an in vitro model of human intestinal epithelial cells, has been demonstrated as the main mechanism driving leukocyte infiltration into the intestine [1]. Leukocyte migration into inflamed tissue is mediated by different groups of adhesion molecules, such as selectins, intercellular and vascular cell adhesion molecules, and integrins, present on both endothelial cells and leukocytes. This allows tissue-specific leukocyte/endothelial interactions, necessary for leukocyte transendothelial migration into tissue [2]. However, in case of a chronic inflammatory reaction, the accumulation of leukocytes is not the result of an adequate reaction to a pathogenic microorganism, but it is a consequence of an unbalanced response that leads to tissue injury and disease, including chronic inflammatory bowel disease (IBD). Therefore, blocking the influx of leukocytes into the intestinal wall may prevent further tissue destruction.

TNF-*α* is elevated in the mucosa of IBD patients and has a main role in the proinflammatory cascade, which induces mucosal-epithelial damage. In particular, multiple lines of evidence indicate that TNF-*α*-induced NF-*κ*B activation actively contributes to the development and maintenance of intestinal inflammation. NF-*κ*B represents a family of transcription factors that are normally kept inactive in the cytoplasm through interaction with inhibitory molecules of the I*κ*B family. In response to multiple stimuli such as inflammatory cytokines, the I*κ*B molecules become phosphorylated by the I*κ*B kinase (IKK) and, as a consequence, free NF-*κ*B enters the nucleus and activates transcription of a variety of genes participating in immune and inflammatory response, cell adhesion, and growth control. NF-*κ*B was found to be activated in mucosal cells of IBD patients, while pharmacological inhibition of NF-*κ*B activity ameliorated intestinal inflammation in mouse models of colitis [3]. Therefore, proper regulation of NF-*κ*B activation at the epithelial interface is crucial for the maintenance of physiological tissue homeostasis and for efficient host defence against environmental insults.

Many investigations reported that plant derivatives, such as polyphenols, possess wide range of biological activities like anti-inflammatory and antioxidant effects. Among the different subclasses of polyphenols, anthocyanins, belonging to the flavonoids group, are the most important health-promoting dietary supplements and are widely distributed in the human diet, with a daily intake between 5 and 215 mg [4]. The bioavailability of anthocyanins is lower than other flavonoids. However, more than 70% of anthocyanins from anthocyanin-rich fruits can reach the colon, which could suggest them as appropriate candidates for supplementation therapy of GI disorders [5]. Anti-inflammatory activity of anthocyanins is mediated by modulation of various inflammatory cytokines or mediators such as IL-1, IL-6, IL-10, TNF-*α*, NF-*κ*B, and COX-2 [6]. In particular, many anthocyanins can affect the activity of the redox-dependent transcription factor NF-*κ*B that modulates critical cell signaling cascades in IBD development [7].

In the present paper, an in vitro coculture system was used to evaluate the capability of TNF-*α*-stimulated intestinal cells to induce vessel endothelial cells activation and the protective effects of cyanidin-3-O-glucoside (the most studied anthocyanin; C3G) against this effect, as well as the underlying mechanisms of action. In order to study the intestinal epithelial barrier function in vitro, it is important to establish and characterize models that will reflect the physiological relationship of cells. To mimic such cell-cell in vitro interactions, a noncontact coculture system was developed to investigate whether differentiated intestinal Caco-2 cells activated with TNF-*α* were able to provide signals that can activate human umbilical endothelial cells (HUVECs). In this model HUVECs are placed in close proximity, but not in contact, to differentiated Caco-2 cells.

## 2. Materials and Methods

### 2.1. Reagents

All the reagents, unless otherwise specified, were purchased from Sigma-Aldrich (Milan, Italy). C3G was supplied from Polyphenols AS, Sandnes, Norway, and was of HPLC grade (>97% purity).

### 2.2. Cell Cultures

*Caco-2 colonic epithelial cell lines*, obtained from the American Tissue Culture Collection (ATCC), were grown in Dulbecco's modified eagle's medium (DMEM) (Sigma-Aldrich) supplemented with 10% fetal bovine serum, 4.0 mM L-glutamine, 1% nonessential amino acids, 100 U/mL penicillin, and 100 *μ*g/mL streptomycin. Cells were incubated at 37°C in a humidified atmosphere with 95% air and 5% CO_2_ and medium was changed every 2 days. Caco-2 cells usually reached confluence 4-5 days after seeding and differentiated into enterocyte-like cells 17–21 days postconfluence. All experiments were carried out with fully differentiated Caco-2 cells. To prepare Caco-2 monolayers, cells were plated at 4 × 10^4^ per cm^2^ on the upper side of transwell inserts (0.4 *μ*m pore size; BD Falcon), grown to confluence, and let to differentiate for 18 days after confluence. Monolayers integrity and formation of tight junctions were assessed by measurement of Transepithelial Electrical Resistance (TEER) by using a Millicell-ERS Voltohmmeter (Millipore, MA, USA). Monolayers used in this study had TEER values ≥ 600 Ω.

*HUVECs* were isolated from freshly obtained human umbilical cords by collagenase digestion of the interior of the umbilical vein as described elsewhere [8] and were cultured in medium 199, supplemented with 20% fetal bovine serum (FBS), 1% L-glutamine, 20 mM HEPES buffer, 100 units/mL penicillin/streptomycin, 50 mg/mL endothelial cell growth factor, and 10 *μ*g/mL heparin, in gelatin pretreated flasks. Cells were maintained in an incubator with humidified atmosphere containing 5% CO_2_ at 37°C. Cells used in this study were from the second to fourth passages.

*Mononuclear cells* have been isolated from human whole blood with Histopaque-1077, following the procedure recommended by the manufacturer. Briefly, heparinized venous blood from healthy donors was centrifuged over Histopaque-1077; the mononuclear cell layer was collected, washed twice with DPBS, suspended in medium 199, and immediately used.

### 2.3. Caco-2-HUVECs Coculture and Treatments

**Figure 1:**
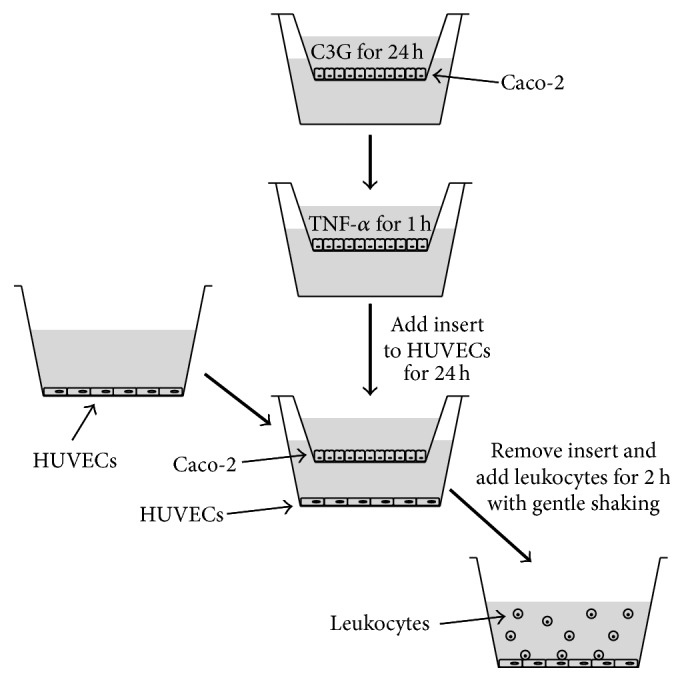
Schematic diagram of coculture assay of differentiated Caco-2 cells and HUVECs and human leukocyte and HUVECS.

In this study, the experimental model described by Maaser et al. [9] with slight modifications was used. Differentiated Caco-2 monolayers, prepared as above described, were pretreated or not with 20 *μ*M C3G for 24 hours. After this time, Caco-2 cell monolayers were washed twice with DPBS, in both the apical and basolateral compartments, and then exposed for 1 hour to 50 ng/mL TNF-*α*. TNF-*α* concentration was chosen on the basis of preliminary experiments indicating that exposure to 50 ng/mL significantly decreased TEER value (15% of reduction) already after 3 hours compared to the untreated control cells. At the end of exposure, Caco-2 cell monolayers were cocultured for 4 hours with HUVECs, previously grown to confluence in 12-well culture plates, by moving the transwell inserts to the multiwells (see Figure 1 for a schematic diagram of the coculture assay). In order to avoid a possible carryover of TNF-*α*, this cytokine was added only in the apical compartment of the transwell inserts and, following Caco-2 cell exposure, inserts were washed three times with DPBS in both the apical and basolateral compartments before being moved over HUVECs. C3G (20 *μ*M), added only to the apical compartment of the transwell, was always freshly dissolved in DMSO and immediately used. The final concentration of DMSO in the culture medium during treatments was <0.1% (v/v). C3G concentrations and exposure time used were consistent with that employed successfully in our previous studies as well as in others [10–12], and within a range physiologically reachable in the digestive tract with a dietary supplementation/intervention [13]. The cell monolayers incubated with 0.1% DMSO in DMEM were used as controls.

In order to support the involvement of NF-*κ*B activation in TNF-*α*-induced inflammation, some experiments were carried out inhibiting NF-*κ*B pathway in Caco-2 cells with the selective IKK inhibitor wedelolactone (WED). In these experiments WED was dissolved in DMSO (DMSO final concentration in the culture medium <0.1%) and added to cell culture media at the final concentration of 25 *μ*M for 1 h before the addition of TNF-*α* according to Ferrari e coll. [10]. The inhibition of the NF-*κ*B pathway under these experimental conditions has been confirmed by western blot, determining p65 levels in Caco-2 cell nucleus (data not shown).

### 2.4. HUVECs-Leukocytes Cocultures and Leukocyte Adhesion

Leukocyte adhesion assay was performed as described elsewhere [14]. Briefly, after the treatments above described, transwell inserts containing Caco-2 monolayers were removed and HUVECs were washed with DPBS and then cocultured with human leukocytes (3 × 10^6^ leukocytes/flask) for 2 h at 37°C with gentle shaking. HUVECs-leukocytes cocultures were visualized under an inverted microscope and photographed using a digital camera. Increase in leukocytes adhesion was calculated in relation to the basal adhesion of leukocytes to control cells that was set to 1 [14].

### 2.5. Nuclear Lysates Preparation

Following appropriate treatment, nuclear lysates were prepared as described elsewhere [15]. Briefly, cells were lysed for 10 min at 2°C in a hypotonic buffer (10 mM Hepes, 1.5 mM MgCl_2_, 10 mM KCl, and 5% glycerol, pH 7.8), containing a cocktail of protease inhibitors (2 *μ*g/mL aprotinin, 1 *μ*g/mL leupeptin, and 1 mM benzamidine) and 1 mM DTT, and treated with 0.65% Igepal (Sigma) for 5 min. Nuclei were recovered by centrifugation at 20,000 ×g for 1 min at 4°C, and nuclear proteins were then obtained by incubating with a hypertonic buffer (20 mM Hepes, 400 mM NaCl, 1 mM MgCl_2_, 0.1 mM EDTA, 1 mM EGTA, and 10% glycerol, pH 7.8) containing protease inhibitors and 1 mM DTT. Nuclear lysates were finally stored at −70°C until use. Purity of nuclear fraction was confirmed by analyzing cytoplasmic marker protein *α*-tubulin (data not shown). Protein concentration in lysates was determined using Bradford reagent.

### 2.6. Immunoblotting

For immunoblot analyses, 40 *μ*g of protein lysates per sample was denatured in 4x SDS-PAGE reducing sample buffer and subjected to SDS-PAGE on 10% acrylamide/bisacrylamide gels. Separated proteins were transferred to nitrocellulose membrane (Hybond-P PVDF, Amersham Bioscience). Residual binding sites on the membrane were blocked with 5% (w/v in TBST) nonfat milk overnight at 4°C. Membranes were then probed with specific primary antibodies, anti-NF-*κ*B p65 rabbit monoclonal antibody (Santa Cruz Biotechnology) (1 : 400) and anti-Lamin B mouse monoclonal antibody (Santa Cruz Biotechnology) (1 : 700), followed by peroxidase-conjugated secondary antibody, anti-rabbit Ig (Cell Signaling Technology) (1 : 5000) and anti-mouse Ig (Cell signaling Technology) (1 : 5000), and visualized with an ECL plus detection system (Amersham Biosciences). The equivalent loading of proteins in each well was confirmed by Ponceau staining and Lamin B control.

### 2.7. Quantitative RT-PCR

Total cellular RNA was isolated with E.Z.N.A® Total RNA kit according to manufacturer's instruction (OMEGA Bio-Tek). After reverse transcription (RT) with oligo (dT)_15_ primers, PCR was performed for identification of TNF-*α*, IL-8, E-selectin, and VCAM-1 mRNA levels. Glyceraldehyde-3-phosphate dehydrogenase (GAPDH) and 18S rRNA were validated as reference genes and they maintained constant expression levels in all conditions and treatments. The specific primers set for the target genes were as follows: 18S rRNA, forward, 5′-GTA ACC CGT TGA ACC CCA TT-3′, reverse, 5′-CCA TCC AAT CGG TAG TAG CG-3′ [16]; GAPDH, forward, 5′-GGC TCT CCA GAA CAT CAT CCC TGC-3′, reverse, 5′-GGG TGT CGC TGT TGA AGT CAG AGG-3′; E-selectin, forward, 5′-CTG CCA AGT GGT AAA ATG TTC AAG-3′, reverse, 5′-TTG GAC TCA GTG GGA GCT TCA-3′ [17]; VCAM-1, forward, 5′-GAA TGG GAG CTC TGT CAC TGT AAG C-3′, reverse, 5′-GAC CAA GAC GGT TGT ATC TCT GGG-3′ [18]; TNF-*α*, forward, 5′-CCA GGC AGT CAG ATC ATC TTC TC-3′, reverse, 5′-AGC TGG TTA TCT CTC AGC TCC AC-3′ [19]; IL-8, forward, 5′-ACT GAG AGT GAT TGA GAG TGG AC-3′, reverse, 5′-AAC CCT CTG CAC CCA GTT TTC-3′ (Primers Bank ID 10834978a2) [20]. Gene expression was assessed by real-time PCR (Applied Biosystem 7300 Real-Time PCR System, Monza, Italy) coupled with the Sybr green JumpStart™ Taq ReadyMix kit. Cycling conditions were a 94°C initial Taq polymerase activation for 2 min, followed by 40 cycles of 94°C denaturation (15 s), and 60°C annealing and extension (1 min). A final dissociation stage was run to generate a melting curve for verification of amplification product specificity. Data were collected and processed with SDS 1.3.1 software (Applied Biosystems, Monza, Italy) and given as threshold cycle (C_t_). The fold increase of mRNA expression, compared with the control cells mRNA expression, was determined using the 2^−ΔΔCt^ method [21].

### 2.8. Statistical Analysis

All the experiments were performed in triplicate and repeated three times. Results are expressed as mean ± SD from three experiments and statistically analyzed by a two-way ANOVA test, followed by Tukey's HSD, using the statistical software ezANOVA (http://www.cabiatl.com/mricro/ezanova/). Differences in groups and treatments were considered significant for *p* < 0.05.

## 3. Results

### 3.1. C3G Prevents TNF-*α*-Induced NF-*κ*B Pathway Activation and Gene Expression of Proinflammatory Cytokines in Caco-2 Cells

TNF-*α* induces a rapid transcription of genes that regulate inflammation, cell survival, proliferation, and differentiation, mainly through the induction of the signaling pathways regulated by NF-*κ*B [10]. It has been hypothesized that activation of NF-*κ*B could represent a key regulator in epithelium-endothelium crosstalk able to induce leukocyte extravasation into mucosal tissues during inflammation. Once activated NF-*κ*B translocates into the nucleus where it binds to promoters of target genes, such as genes codifying for proinflammatory proteins.

**Figure 2:**
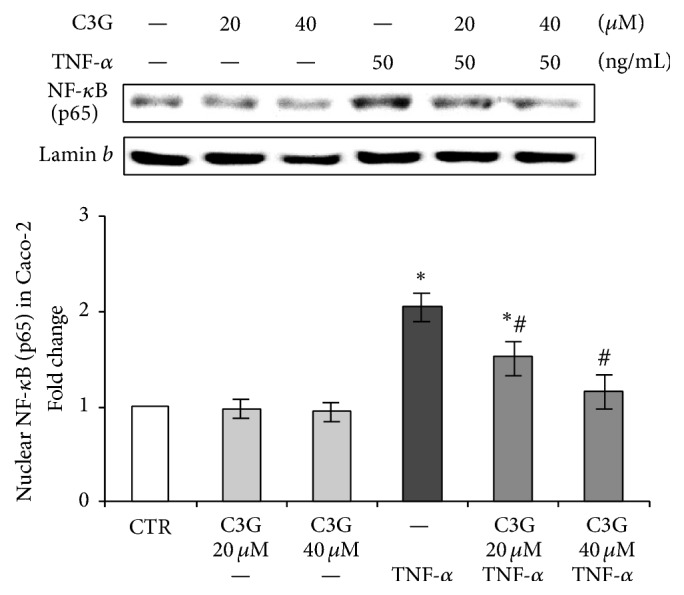
Nuclear NF-*κ*B (p65) in Caco-2 cells. The Caco-2 monolayer was pretreated for 24 hours with C3G (20 or 40 *μ*M) and subsequently exposed to 50 ng/mL TNF-*α* for 1 hour. Cultures treated with the vehicle alone (0.1% DMSO) were used as controls (CTR). Caco-2 cell nuclear lysates were analyzed by western blot, and nuclear localization of the p65 protein was evaluated. Results are reported as fold change against control and expressed as mean ± SD of three independent experiments. NF-*κ*B (p65) intensity values were normalized to the corresponding Lamin *b* value. ^*∗*^*p* < 0.05 versus CTR; ^#^*p* < 0.05 versus TNF-*α*.

The translocation of NF-*κ*B into the nucleus was evidenced by the increased levels of p65 subunit in the nuclear extracts of Caco-2 cells treated with 50 ng/mL TNF-*α* for 1 h, whereas C3G pretreatment prevented, in a dose-dependent way, TNF-*α*-induced nuclear translocation of p65 (Figure 2). Treatment with C3G alone, however, had no effect on the nuclear localization of NF-*κ*B. According to these results, the lower C3G concentration (20 *μ*M) able to reduce p65 nuclear translocation was used for all the subsequent experiments. Furthermore, 20 *μ*M is within a range considered physiologically reachable in the gut with a diet with anthocyanin-rich foods or with a pharmacological intervention [22].

TNF-*α* and IL-8 are among the proinflammatory genes having a *κ*B site in their promoter and thus regulated by NF-*κ*B. TNF-*α* is a cytokine able to increase the expression of proinflammatory cytokines, chemokines, adhesion molecules, and other inflammatory mediators [23]. This cytokine is known to induce endothelial dysfunction in HUVECs [24, 25] and, as hypothesized by Maaser and coll. [9], is one of the main candidates responsible for epithelial-endothelial communication. IL-8 is a chemokine recruiting neutrophils into mucosa and present in both enterocytes and mucosal inflammatory cells [26, 27], and its production is induced by TNF-*α* in Caco-2 cells [28].

**Figure 3:**
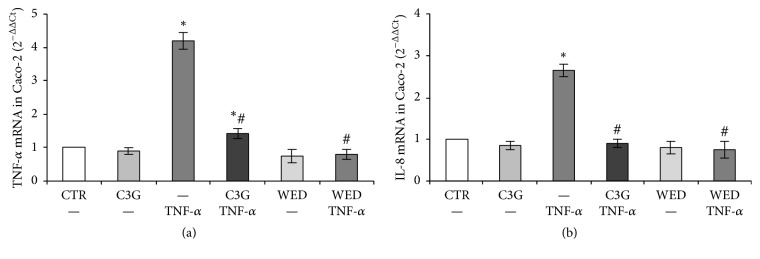
(a) TNF-*α* and (b) IL-8 gene expression in Caco-2 cells. The Caco-2 cell monolayer was pretreated for 24 hours with 20 *μ*M C3G or with 25 *μ*M WED for one hour and subsequently exposed to 50 ng/mL TNF-*α* for one hour. Cultures treated with the vehicle alone (0.1% DMSO) were used as controls (CTR). Results, deriving from three independent experiments, are expressed as 2^−ΔΔCt^ (mean ± SD). 18S rRNA was used as housekeeping gene. ^*∗*^*p* < 0.05 versus CTR; ^#^*p* < 0.05 versus TNF-*α*.

In this experimental model Caco-2 exposure for one hour to 50 ng/mL TNF-*α* induced the expression of both the proinflammatory genes (Figure 3). Caco-2 monolayer pretreatment with 20 *μ*M C3G prevented TNF-*α*-induced gene expression of TNF-*α* (Figure 3(a)) and IL-8 (Figure 3(b)); in particular, IL-8 expression levels were similar to those found in control cells (Figure 3(b)). Treatment with C3G alone did not affect basal levels of TNF-*α* and IL-8 gene expression (Figure 3).

Since these two proinflammatory genes are regulated by NF-*κ*B pathway, some experiments were carried out inhibiting NF-*κ*B pathway in Caco-2 cells with WED, a selective pharmacological inhibitor of IKK, which is an upstream kinase critical for activation of NF-*κ*B. As shown in Figure 3, selective inhibition of NF-*κ*B pathway with WED inhibited TNF-*α* and IL-8 gene expression.

### 3.2. C3G Inhibits Endothelial Activation in HUVECs Cocultured with TNF-*α*-Stimulated Caco-2

Intestinal epithelium is the first line of defence against enteric pathogens and toxins [29] and is located in close proximity to the intestinal microvascular endothelium, thus representing the final anatomic border before host microcirculation, so it is likely that intestinal epithelial alterations may influence the adjacent cells. Endothelial cells are the major constituent of the microvasculature that line blood vessels. Following intestinal epithelium inflammation, endothelial cells undergo rapid and remarkable changes in response to elevated levels of proinflammatory cytokines and chemokines [30]. In the present study we hypothesized that NF-*κ*B-modulated activation of proinflammatory mediators in epithelial cells may influence endothelial activation.

To mimic such cell-cell interactions in vitro we used a coculture system that places endothelial cells in close proximity, but not in contact, to epithelial cells. In this model we evaluated if TNF-*α*-stimulated Caco-2 cells were able to induce endothelial activation in HUVECs, with the purpose of studying a possible direct role of intestinal epithelial cells in leukocyte recruitment mediated by adhesion molecules.

With this aim differentiated Caco-2 monolayers, grown on the upper side of transwell inserts, were exposed for 1 hour to 50 ng/mL TNF-*α*, and then placed for the next 4 hours in close proximity to HUVECs grown on the bottom of the wells of 12 wells multiwells plates (see Figure 1 for details).

**Figure 4:**
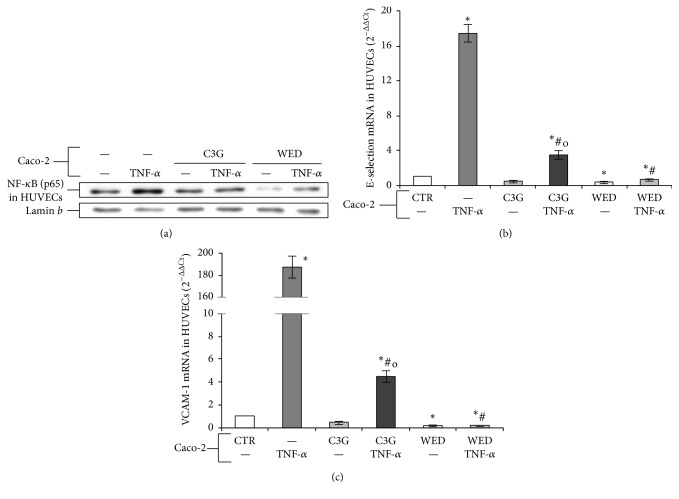
(a) Nuclear NF-*κ*B (p65) localization and gene expression of E-selectin (b) and VCAM-1 (c) in HUVECs. HUVECs were cocultured for 4 hours with Caco-2 cell monolayers previously pretreated for 24 hours with 20 *μ*M C3G or with 25 *μ*M WED for one hour and subsequently exposed to 50 ng/mL TNF-*α* for one hour. Cultures treated with Caco-2 cells exposed to the vehicle alone (0.1% DMSO) were used as controls (CTR). (a) HUVEC nuclear lysates were analyzed by western blot, and nuclear localization of the p65 protein was evaluated. (b-c) HUVEC E-selectin and VCAM-1 RNA were analyzed by RT-PCR and data are expressed as 2^−ΔΔCt^; GAPDH was used as housekeeping gene. Results, deriving from three independent experiments, are reported as mean ± SD. ^*∗*^*p* < 0.01 versus CTR; ^#^*p* < 0.01 versus TNF-*α*; ^o^*p* < 0.01 versus C3G.

Caco-2 cells exposed to TNF-*α* induced HUVECs activation, as evidenced by the increased levels of p65 in HUVEC nuclear extracts (Figure 4(a)) and by an upregulation of gene expression of the adhesion molecules E-selectin and VCAM-1 (Figures 4(b) and 4(c)), presenting the active motif recognized by NF-*κ*B in their promoter. Interestingly, Caco-2 cells pretreatment with 20 *μ*M C3G for 24 hours reduced NF-*κ*B nuclear translocation, as well as activation of adhesion molecules gene expression, induced by TNF-*α* in HUVECs (Figure 4). C3G pretreatment does not significantly affect NF-*κ*B and adhesion molecules with value similar to control.

Furthermore, our data demonstrated that also WED inhibition of NF-*κ*B pathway in Caco-2 cells exposed to TNF-*α* prevented endothelial activation (Figure 4). These results further support the hypothesis that the inhibition of the NF-*κ*B pathway in the intestinal TNF-*α*-exposed Caco-2 cells could represent a potential mechanism by which C3G exerts its protective effects against the subsequent epithelial-induced endothelial activation.

### 3.3. C3G Inhibits Leukocyte Adhesion in HUVECs Cocultured with TNF-*α*-Stimulated Caco-2

**Figure 5:**
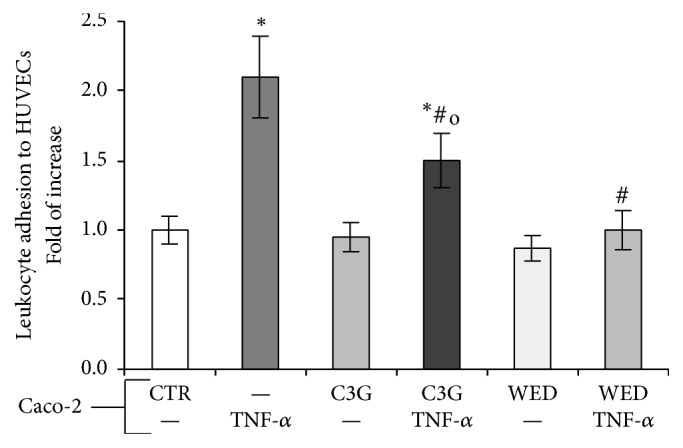
Leukocyte adhesion in HUVECs cocultured for 4 hours with Caco-2 cell monolayers previously pretreated for 24 hours with 20 *μ*M C3G or with 25 *μ*M WED for one hour and subsequently exposed to 50 ng/mL TNF-*α* for one hour. Increase in leukocyte adhesion is expressed as fold of change versus the basal adhesion of leukocytes to control (HUVECs treated with Caco-2 exposed to the vehicle alone; CTR) that was set to 1. Data are reported as mean (±SD) of three separate experiments. ^*∗*^*p* < 0.01 versus CTR; ^#^*p* < 0.01 versus TNF-*α*; ^o^*p* < 0.01 versus C3G.

The expression of adhesion molecules is responsible for leukocytes adhesion to vascular endothelium and their migration into subendothelial space. In order to confirm the inhibitory activity of C3G on endothelial cells induced by TNF-*α*-stimulated Caco-2 cells, after the exposure to Caco-2 cells, HUVECs were cocultured with isolated human leukocytes as described in Section 2.4.  Figure 5 shows that the number of leukocytes adhered to the endothelial cells exposed to TNF-*α*-stimulated Caco-2 cells was higher than that observed in controls, but it appeared to be reduced by Caco-2 cells pretreatment with C3G, demonstrating that this anthocyanin is able to prevent, in Caco-2 cells exposed to TNF-*α*, the release of proinflammatory mediators able to induce endothelial activation. Also in this case, NF-*κ*B inhibition in Caco-2 cells by WED reduced leukocytes adhesion to HUVECs.

## 4. Discussion

The intestinal epithelium has a strategic position as a protective physical barrier to luminal damaging agents and actively contributes to the mucosal immune system. It lies in proximity to a number of subepithelial cells, including vascular endothelium, so influencing the immunological response of the gut. Several proinflammatory cytokines, such as TNF-*α* and interferon-*γ*, have been shown to induce activation of the mucosal immune system, resulting in sustained inflammation and tissue damage. Furthermore, the polarized basolateral secretion of inflammatory mediators, such as IL-8 and other CXC chemokines, from gut epithelial cells followed by activation of NF-*κ*B signaling and inflammatory pathways in endothelial cells, has been found to efficiently trigger extravasation of neutrophils from the vasculature.

Neutrophil transepithelial migration plays a major role in mucosal-epithelial defence in inflammatory diseases. Leukocytes migrate to their sites of activity via a carefully regulated multistep adhesion cascade. Activated neutrophils first adhere to the endothelial cells that line the blood vessels and then migrate across the endothelium and through the extracellular matrix to arrive at effector sites within the tissues. The exit sites for leukocyte emigration are the postcapillary venules, which are lined with endothelial cells expressing ligands (e.g., VCAM-1 and E-selectin) that facilitate leukocyte adhesion.

In this paper we aimed to evaluate the effects of TNF-*α*-stimulated intestinal cells on endothelial cells activation by using a coculture in vitro system, as well as the protective effects of C3G Caco-2 cell pretreatment on the TNF-*α*-induced alterations in epithelial cells and on the subsequent endothelial dysfunction.

Our results showed that 1 h of TNF-*α* exposure induced nuclear translocation of NF-*κ*B in Caco-2 cells, whereas C3G pretreatment reduced dose-dependently this effect. The present data are in accordance with other previous studies reporting the inhibitory activity of anthocyanins on the NF-*κ*B proinflammatory pathway in Caco-2 cells [10, 31, 32]. Furthermore, in vivo studies demonstrated that feeding pigs with an anthocyanin-rich extract reduces transactivation of NF-*κ*B in the duodenal mucosa and in turn lowers transcript levels of various NF-*κ*B target genes involved in the inflammatory process [33].

Furthermore, we observed that Caco-2 cell exposure to TNF-*α* induced the expression of two genes coding for the proinflammatory mediators TNF-*α* and IL-8 (Figure 3). Also in this case, C3G pretreatment reduced TNF-*α* and IL-8 mRNA levels induced by TNF-*α* in Caco-2 cells. As TNF-*α* and IL-8 genes contain a binding site for the inducible transcription factor NF-*κ*B, we hypothesize that the C3G protective effects are attributable to the inhibition of NF-*κ*B pathway. Interestingly, the selective pharmacological NF-*κ*B inhibitor WED was able to totally inhibit the overexpression of the TNF-*α* and IL-8 genes induced by TNF-*α*, thus supporting that the NF-*κ*B inhibition may be the main mechanism in the protective effect of C3G. NF-*κ*B was demonstrated as main molecular target involved in C3G protective effect also in other in vitro and in vivo experimental models [22]. Furthermore, numerous studies provided evidence that anthocyanins activation of a redox-sensitive gene-regulatory network mediated by the NF-E2-related factor-2 (Nrf2) is intimately involved in reducing NF-*κ*B pathway [10, 34].

TNF-*α* and IL-8 are the main proinflammatory mediators released by intestinal cells, involved in recruitment of leukocytes from microcirculation. In case of a chronic inflammatory reaction, the accumulation of leukocytes is not the result of an adequate reaction to a pathogenic microorganism but it is the result of an unbalanced reaction that leads to tissue damage and disease, such as chronic IBD [35]. So, we evaluated changes in endothelial cells exposed to activated Caco-2 cells. Our data demonstrate that Caco-2 cells exposed to TNF-*α* induced endothelial activation as observed by increased E-selectin and VCAM-1 mRNA and NF-*κ*B levels in HUVECs (Figure 4). Also in this case, C3G pretreatment of TNF-*α*-stimulated Caco-2 cells reduced E-selectin and VCAM-1 gene expression and NF-*κ*B nuclear translocation in HUVECs. Furthermore, we observed that inhibition of NF-*κ*B pathway by WED in TNF-*α*-stimulated Caco-2 cells was able to reduce adhesion molecule mRNA and NF-*κ*B nuclear translocation in HUVECs. These effects were confirmed also at endothelial functional level. In fact, TNF-*α*-stimulated Caco-2 cells induced increased leukocyte adhesion to HUVECs, while C3G pretreatment of Caco-2 reduced this effect. These data confirmed that the increased production of chemokines from intestinal epithelial cells in response to inflammatory stimuli, involved in endothelial leukocyte recruitment, can be modulated by anthocyanins. However, our results support a specific mechanism for C3G protective effects. Interestingly, Caco-2 exposure to WED restored leukocyte recruitment to value similar to control Caco-2 cells, thus demonstrating that C3G modulation of intestinal NF-*κ*B pathway is crucial in epithelial-endothelial interactions.

## 5. Conclusions

In conclusion our findings show that C3G is able to counteract the acute proinflammatory effects of TNF-*α* in intestinal epithelium and to reduce leukocyte recruitment from microcirculation. Furthermore, C3G alone did not alter physiological properties of Caco-2 cells. Selective inhibition of the NF-*κ*B pathway in epithelial cells represents the main mechanism by which this anthocyanin exerts its protective effect. This hypothesis is strongly supported by our previous data reporting the existence of a crosstalk between the NF-*κ*B pathway and the redox-dependent Nrf2 pathway which may be induced by C3G [11, 36]. Our data suggest that anthocyanins could contribute, as a complementary or preventive approach, to the management of chronic gut inflammatory diseases.

## Abbreviations

C3G: Cyanidin-3-O-glucoside
HUVECs: Human umbilical vein endothelial cells
WED: Wedelolactone.
